# Association between socio-economic status and non-communicable disease risk in young adults from Kenya, South Africa, and the United Kingdom

**DOI:** 10.1038/s41598-023-28013-4

**Published:** 2023-01-13

**Authors:** Asanda Mtintsilana, Ashleigh Craig, Witness Mapanga, Siphiwe N. Dlamini, Shane A. Norris

**Affiliations:** 1 SA MRC/Wits Developmental Pathways for Health Research Unit, Department of Paediatrics, Faculty of Health Sciences, School of Clinical Medicine, University of the Witwatersrand, Private Bag X3, Johannesburg, 2050 South Africa; 2 Noncommunicable Diseases Research Division, Wits Health Consortium (PTY) Ltd, Johannesburg, South Africa; 3 DSI-NRF Centre of Excellence in Human Development, University of the Witwatersrand, Johannesburg, Gauteng South Africa; 4 Global Health Research Institute, School of Human Development and Health, University of Southampton, Southampton, UK

**Keywords:** Diseases, Risk factors

## Abstract

There is a pressing need for global health preventions to curb the escalating burden of non-communicable diseases (NCDs). Utilising multi-country study designs can improve our understanding of how socio-economic context shapes the aetiology of NCDs, and this has great potential to advance global health interventions. We examined the association between socio-economic status (SES) and NCD risk, and the potential confounding effects of smoking and alcohol intake in young adults (18–35-year-olds) from Kenya, South Africa (SA), and the United Kingdom (UK). Our study was a cross-sectional online survey that included 3000 respondents (n = 1000 per country, 50% women) conducted in April 2022. We utilised information on twelve NCDs to classify respondents as having “no condition”, “one condition”, and “multimorbidity” (having two or more conditions). A total household asset score was calculated and used as a proxy of SES, and subsequently categorised into quintiles (Q1–Q5; lowest-highest). Ordered logistic regression was used to test the associations between NCD risk and exposure variables. In the UK sample, we found that those in the second lowest SES quintile (Q2) had lower odds of developing NCDs than their lowest SES counterparts (Q1). In contrast, South African and Kenyan youth with a SES score between middle and highest quintiles (Q3–Q5) were more likely to develop NCDs than the lowest SES quintile group. In all countries, smoking and/or alcohol intake were associated with higher odds of developing NCDs, and showed some confounding effects on the SES-NCD relationships. Specifically, in Kenya, the risk of developing NCD was more than two times higher in those in the middle (Q3) SES group (OR 2.493; 95% CI 1.519–4.091; p < 0.001) compared to their lowest (Q1) SES counterparts. After adjusting for smoking and alcohol, the ORs of middle (Q3) SES group changed from 2.493 to 2.241 (1.360–3.721; p = 0.002). Overall, we found that the strength and direction of SES-NCD associations differed within and between countries. This study highlights how different SES contexts shape the risk of NCDs among young adults residing in countries at different levels of economic development.

## Introduction

Non-communicable diseases (NCDs) such as type 2 diabetes, cardiovascular disease (CVD), and cancer are the leading cause of mortality, accounting for over 71.0% of all global deaths^1,2^. Regrettably, 77.0% of all NCD-related deaths occur in low- and middle-income countries (LMICs)^2^. The factors underlying the high burden of mortality in LMICs are multi-faceted including population growth, ageing, urbanisation, and dearth of knowledge and awareness about NCDs^3–5^. Other factors include the co-existence of NCDs with infectious diseases which overburdens the fragile healthcare systems^6^. Additionally, there is also inadequate funding to address NCDs in comparison to infectious diseases^7,8^, which aggravates the prevalence of NCDs and related deaths. Relevant to this study is that LMICs are also battling to provide effective preventative and treatment strategies and policies to combat the rapid increase in NCDs and their prominent risk factors^3,9^. Behavioural risk factors such as harmful use of alcohol and tobacco are one of the major drivers of NCDs^9–12^. Given that these behavioural risk factors are often adopted earlier in life^9–12^, understanding the aetiology of NCDs in young adults (18–35 years of age) provides a window period for behaviour modification for the prevention of NCDs and related deaths. This is paramount since an estimated 1.7 million deaths occur in people younger than 30 years of age^1,2^.

Although the aetiology of NCDs is multi-factorial, there is growing interest in the association between socio-economic status (SES) and the risk of developing NCDs^9,13^. SES includes several indicators such as affluency (i.e., owning assets such as a motor vehicle), education attained, and economy, which represent the SES of an individual or the socio-economic development of a country^9,14,15^. Individuals or communities with low SES are typically at greater risk of developing NCDs compared to their counterparts^9,14,15^. However, large-scale studies often focus on the association between SES and NCD risk at a national level^9,14,15^. This approach, however, fails to recognise that regardless of the country’s income status, there might be individual variations in the strength and direction of this association at different levels of SES.

It is well established that the epidemiology of NCDs differs between high-income countries (HICs) and LMICs^16–19^. For instance, SES is inversely associated with NCDs such as obesity, type 2 diabetes, and hypertension in HICs^15–17^, however, this relationship tends to be positive in LMICs^18–20^. Although the mechanisms underlying these discrepancies are not fully understood, several risk factors including population growth, ageing, and urbanisation and epidemiological transition have been implicated in the rising prevalence of NCDs, particularly in LMICs^3,4^. Accordingly, urbanisation and the epidemiological transition are at different stages within the African region^21^, with South Africa (SA) being farthest along the transition^21^. This may partly explain why SA has reportedly higher NCD prevalence (e.g., obesity and hypertension) and associated risk factors than its counterparts^5,22–24^. Notably, these studies were conducted in middle-age and older participants^5,22–24^, yet a better understanding of the adverse socio-environmental factors on NCD epidemiology during early life is equally crucial for combating NCDs in the African region.

Given the global health concern around the increasing burden of NDCs and their detrimental effects on human development and public health systems, multi-country comparative analyses and collaborations are essential for the development of effective strategies to curb the rising prevalence of NCDs, especially if established earlier in life. Therefore, the study aims were to examine the association between SES and NCD incidence, and the potential confounding effects of smoking and alcohol intake in young adults (18–35-year-olds) from three countries: lower-middle-income country (Kenya); upper middle-income country (SA) and high-income country (United Kingdom (UK)).

## Methods and materials

### Survey integrity and processes

**Figure 1:**
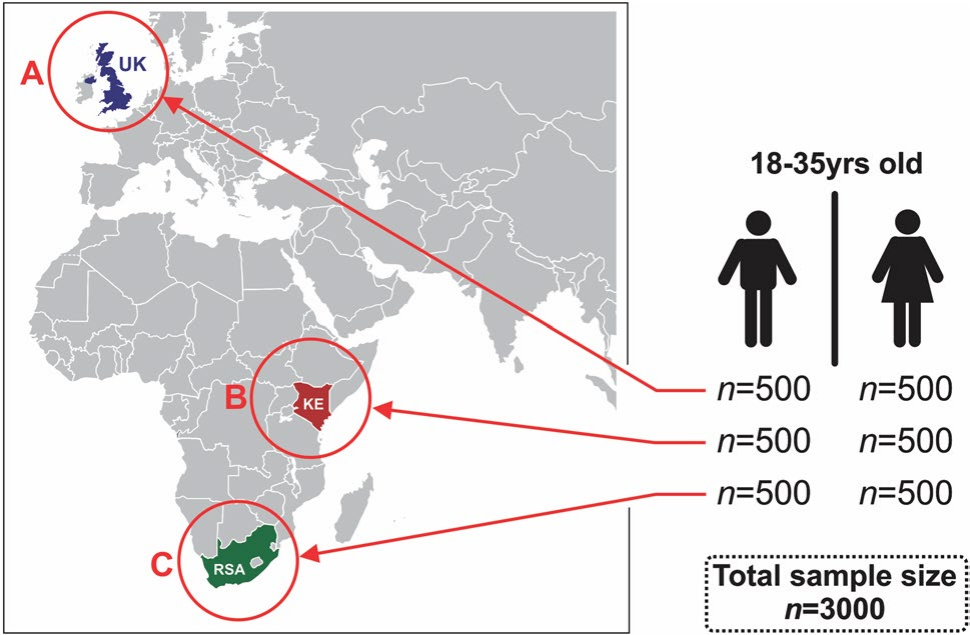
Geographical representation of the three countries in which the survey was conducted. *KE* Kenya, *RSA* Republic of South Africa, *UK* United Kingdom.

This cross-sectional study was conducted in April 2022 using “i-Say panel” as outlined in Supplementary Figure S1. Briefly, prospective panellists or people interested in participating in survey were recruited via a multi-source recruitment process that meet the quality of Global Research Standards. Through a partnership with an American consumer credit reporting agency (TransUnion), digital fingerprinting technology known as TruValidate collected the respondents’ device information. Thereafter, a unique identifier was issued to the respondents’ device, based on its distinctive characteristics. Using various authentication tools (e.g., Multi-Factor authentication), the device underwent a fraud check and deduplication review to prevent the creation of multiple entries and panel accounts in the same survey. Additionally, a tool known as Dataguard is used, which is a cyber robot designed to detect open ends (e.g., copied and pasted text and exaggerated and/or unrealistic typing speed), data pattern recognition (algorithms to review new panellists and flag those that match the profile of known fraudsters), and straight lining (identifies respondents who gave the same answer to all statements in a grid question). Furthermore, this tool measures the pace of survey completion in answers per minute (speeding) and ensures that respondents who do not answer questions meaningfully or click through the survey randomly to earn rewards are removed. Therefore, Dataguard protects against suspicious respondents and ensures that respondents are valid and engaged appropriately with the survey. If the respondents are caught by Dataguard, they are flagged and automatically removed from the survey in real-time. The survey was concluded when 1000 valid respondents from each country (UK, SA, and Kenya) completed the survey and deemed valid through the backend checks described above. Therefore, the sample (n = 3000) was not randomly recruited, rather targeted. Furthermore, the panel was recruited to be nationally representative of the country’s 18–35-year-olds with internet access, thus may not necessarily be representative of the entire youth or general population. In each country, an equal ratio of males and females was sampled (Fig. 1).

### Survey data collected

An online survey questionnaire was administered through Ipsos, measuring socio-demographic characteristics (e.g., household assets), medical history (e.g., NCDs), and lifestyle behavioural risk factors (smoking and alcohol intake) (Supplementary file 2). A household asset score from a list of 22 assets was used as an indicator of affluence (furniture, mattress, bed, gas cooker, stove, refrigerator, air conditioner, washing machine, bicycle, motorbike, vehicle, generator, fan, microwave, television, computer or tablet, satellite dish, smartphone, mobile phone, flush toilet, tap water, and electricity). This score was based on standard measures used in the Demographic and Health Surveys household questionnaire (www.measuredhs.com). Data on twelve NCDs (heart attack, stroke, high cholesterol, diabetes, overweight or obesity, asthma or chronic obstructive pulmonary disease, sore joints or muscle problems, cancer, mental health condition (e.g., depression or anxiety), liver disease, chronic kidney disease, and hypertension) were collected. Respondents were asked whether they had been told by a doctor or any healthcare professional (e.g., nurse or community health worker) if they had any of the above disease(s). If respondents replied “no” to all twelve conditions, they were classified as having “No condition”. In contrast, respondents who answered “yes” to one or two or more conditions were classified as having “one condition” or “multimorbidity”, respectively. Current smoking status (yes or no) was measured, with smoking frequency (amount per day) recorded and categorised into four groups, based on scoring in the range 0–1; 1–5; 6–10; and 11 or more. Notably, smoking was not limited to cigarettes but also included chewing tobacco, pipe, cigars, vape, hubbly, and snuff or dagga. Furthermore, participants were asked if they consumed alcohol (yes or no), and those who replied “yes” were further asked to estimate their frequency which was also categorised into four groups, occasionally/not every week; once a week; 2–3 times a week; and every day.

### Statistical analysis

Where applicable, data were tested for normality using distribution plots or the Shapiro Wilk test. Data are presented as frequencies (%) or median (interquartile range). Differences between the three countries were compared using the Kruskal–Wallis test. The Chi-square test of independence was used for the categorical variables. For each country, the sum of 22 household items (a proxy of SES) was categorised into quintiles [quintile 1 (Q1) (lowest) to quintile 5 (Q5) (highest)] and used as an exposure variable in the subsequent analyses. The quintiles were created using the xtile command on Stata^®^, which was generated three times. Therefore, in each country respondents in quintile 1 have the lowest SES score (“poor”), while those in quintile 5 have the highest SES score (“wealthy”). Four ordered logistic regression analyses were performed to examine the effects of SES, smoking, and alcohol intake in driving NCDs (a 3-level categorical outcome variable, with “no condition” as the reference group). The first model tested the association between SES and NCD. The second model, which was divided into two parts, explored the effects of smoking (model 2.1) and alcohol intake (model 2.2) on NCD risk. Both smoking and alcohol intake were assessed as five-level confounder variables with non-smokers and non-drinkers as reference groups. Thereafter, the third model was performed to test the hypothesis that smoking (model 3.1), or alcohol intake (model 3.2) will reduce the odds ratios (OR) and/or significance (p value) of SES-NCD association. For these analyses, both confounders (smoking or alcohol) and exposure (SES) variables were included in the same models. In the final model, the two confounders were included in the same model to assess the independent effect of SES on NCD outcome. Two sets of results were generated by stratifying the outputs according to country and combining the three countries into one sample (combined analysis). Notably, the three counties were combined to examine the pooled overall effect of the SES–NCD relationship in the entire sample. All models were controlled for the potential effect of age, gender, and country (in combined sample only). All data analyses were conducted using Stata^®^ (Version 17.0, StataCorp, College Station, TX, USA). A two-tailed test was considered statistically significant if p < 0.050.

### Ethical approval

As a market research house, IPSOS and its entities do not have to obtain ethical approval for their panels as they are members of research institutions and have to comply to the industry standards and regulations set out by such institutions including the South African Market Research Association, the European Society for Opinion and Market Research (ESOMAR), and Protection of Personal Information Act in South Africa. Kenya adheres to ESOMAR and the UK adheres to the Market Research Society and General Data Protection Regulation regulations. Also, panel surveys are opt-in surveys, meaning respondents choose to become a member understanding the data privacy policy and confidentiality procedures. However, any research study that involves human participants does require ethics approval, which we obtained from the University of Witwatersrand Human Ethics Research Committee (Non-Medical) (ethics clearance certificate number: H21/06/36). All eligible members of the panel population were sent an invitation to participate and the information sheet about the study. Respondents who were willing to participate in the survey provided consent and completed the survey.

## Results

### Socio-demographic, behavioural, and non-communicable disease characteristics in the total sample and various countries

Due to the nature of the survey which targets 1000 participants completing the survey per country, no data was missing. Further post-survey data checking removed eight respondents from the 3000 due to their indiscriminate and unlikely reply (yes) to all twelve listed NCDs. There were significant differences in age group proportions between the three countries (p < 0.001) (Table 1). Notably, the UK had the highest percentage of young adults within the 30–35 years of age (47.7%). As expected, household SES (as a total score or country-quintile categorical variable) differed between the three countries. South Africa had the highest percentage of smokers (36.9%) and those who consume alcohol (71.5%). Six out of twelve NCDs, namely, heart attack, high cholesterol, overweight or obesity, chronic lung diseases, mental health conditions, and hypertension were significantly different between the countries (p < 0.05) (Table 1). For example, the UK had a higher mental health condition prevalence (37.7%) than both SA (26.3%) and Kenya (27.9%) (p < 0.001). In contrast, SA (15.2%) and Kenya (15.5%) had a higher hypertension incidence than the UK (9.9%) (p < 0.001). Notably, the proportion of participants that had “no condition”, “one condition”, or multimorbidity differed between the three countries.

**Table 1:** Socio-demographic, behavioural, and non-communicable disease characteristics of young adults from United Kingdom, South Africa, and Kenya.

	Combined sample	United Kingdom	South Africa	Kenya	P value
Age groups (years) (n (%))					
18–21	439 (14.6)	122 (12.2)	171 (17.1)	146 (14.6)	< 0.001
22–25	797 (26.6)	185 (18.5)	270 (27.0)	342 (34.2)	
26–29	772 (25.7)	216 (21.6)	260 (26.0)	296 (29.6)	
30–35	992 (33.1)	477 (47.7)	299 (29.9)	216 (21.6)	
Household SES (sum of 22 items)	14 (11–16)	15 (13–17)	15 (13–17)	12 (9–15)	< 0.001
Household SES in quintiles (n (%))					
Q1 (Lowest)	747 (24.9)	273 (27.3)	240 (24.0)	234 (23.4)	< 0.001
Q2 (Second lowest)	635 (21.2)	137 (13.7)	263 (26.3)	235 (23.5)	
Q3 (Middle)	655 (21.8)	317 (31.7)	155 (15.5)	183 (18.3)	
Q4 (Upper middle)	486 (16.2)	109 (10.9)	222 (22.2)	155 (15.5)	
Q5 (Highest)	477 (15.9)	164 (16.4)	120 (12.0)	193 (19.3)	
Behavioural factors (%)					
Currently smoking (yes)	835 (27.8)	296 (29.6)	369 (36.9)	170 (17.0)	< 0.001
Smoking frequency amongst the 27.8% who reported smoking (n, %)					
Less than 1 per day	166 (19.9)	52 (17.6)	57 (15.4)	57 (33.5)	< 0.001
1–5 per day	412 (49.3)	124 (41.9)	189 (51.2)	99 (58.2)	
6–10 per day	162 (19.4)	64 (21.6)	90 (24.4)	8 (4.7)	
11 or more per day	95 (11.4)	56 (18.9)	33 (8.9)	6 (3.5)	
Alcohol intake (yes)	1851 (61.7)	627 (62.7)	715 (71.5)	509 (50.9)	< 0.001
Alcohol intake frequency among the 61.7% that reported alcohol consumption (n (%))					
Occasionally/not every week	902 (48.7)	249 (39.7)	387 (54.1)	266 (52.3)	< 0.001
Once a week	477 (25.8)	198 (31.6)	149 (20.8)	130 (25.5)	
2 to 3 times a week	438 (23.7)	162 (25.8)	172 (24.1)	104 (20.4)	
Everyday	34 (1.8)	18 (2.9)	7 (1.0)	9 (1.8)	
Non-communicable diseases (n (%))					
Heart attack	133 (4.4)	15 (1.5)	58 (5.8)	60 (6.0)	< 0.001
Stroke	39 (1.3)	19 (1.9)	9 (0.9)	11 (1.1)	0.115
High blood cholesterol	275 (9.2)	71 (7.1)	112 (11.2)	92 (9.2)	0.006
Diabetes	220 (7.3)	72 (7.2)	72 (7.2)	76 (7.6)	0.927
Overweight or obesity	585 (19.5)	224 (22.4)	159 (16.0)	202 (20.2)	0.001
Asthma/Chronic lung disease	398 (13.3)	161 (16.1)	128 (12.8)	109 (10.9)	0.003
Sore joints or muscle problems	497 (16.6)	148 (14.8)	174 (17.4)	175 (17.5)	0.174
Cancer	36 (1.2)	14 (1.4)	11 (1.1)	11 (1.1)	0.781
Mental health conditions	918 (30.7)	377 (37.7)	262 (26.3)	279 (27.9)	< 0.001
Liver disease	36 (1.2)	11 (1.1)	9 (0.9)	16 (1.6)	0.337
Chronic kidney disease	36 (1.2)	12 (1.2)	12 (1.2)	12 (1.2)	1.000
Hypertension (high blood pressure)	405 (13.5)	99 (9.9)	151 (15.2)	155 (15.5)	< 0.001
Non-communicable disease category (n (%))					
No condition	138 (4.6)	8 (0.8)	76 (7.6)	54 (5.4)	< 0.001
One condition	2404 (80.3)	843 (84.3)	777 (77.7)	784 (78.4)	
Multimorbidity	450 (15.0)	148 (14.8)	142 (14.2)	160 (16.0)	

Data are presented as frequencies (%) and median (interquartile range).

### The effect of SES on non-communicable diseases risk, with smoking and alcohol intake as confounders in youth from United Kingdom, South Africa, and Kenya

Table 2 shows the series of ordered regression outputs, whereby SES and behavioural risk factors (smoking and alcohol intake) were regressed on NCD category. In the UK sample, those who were in the second lowest SES quintile (Q2) were 46.7% less likely to develop NCDs than their lowest (Q1) SES counterparts. However, there were no differences in NCD risk between Q1 and upper quintile groups (i.e., Q3, Q4, and Q5) (Model 1). Furthermore, respondents that reported a smoking frequency of 11 or more cigarettes per day had a twofold higher of developing NCDs than their counterparts (Model 2.1). In contrast, alcohol intake was not associated with the risk of developing an NCD in the UK group (Model 2.2.). Adjustments made for smoking and alcohol intake had only minor effects on the influence of SES on NCD risk (Table 2). For example, the risk of developing NCD in those in Q2 declined from model 1 [odds ratios (ORs) per unit increase in SES quintile level: 0.533; 95% confidence interval (CI) 0.288–0.985; p = 0.045] to model 4 (0.506; 0.272–0.943; p = 0.032) after controlling for the potential behavioural effects (Table 2).

**Table 2:** Ordered logistic regression analyses for the effect of SES on non-communicable diseases risk, with smoking and alcohol intake as moderators in young adults from United Kingdom, South Africa, and Kenya.

	United Kingdom		South Africa		Kenya		Combined sample	
	OR (95% CI)	P value	OR (95% CI)	P value	OR (95% CI)	P value	OR (95% CI)	P value
Model 1 (Main exposure variable only)								
Household SES in quintiles								
Q1 (Lowest)	Ref	Ref	Ref	Ref	Ref	Ref	Ref	Ref
Q2 (Second lowest)	0.53 (0.29–0.98)	0.045	1.50 (0.97–2.32)	0.069	1.48 (0.92–2.38)	0.102	1.12 (0.84–1.48)	0.435
Q3 (Middle)	0.82 (0.53–1.27)	0.372	2.06 (1.25–3.40)	0.005	2.49 (1.52–4.09)	< 0.001	1.61 (1.23–2.11)	0.001
Q4 (Upper middle)	0.57 (0.30–1.11)	0.098	2.23 (1.42–3.52)	0.001	2.19 (1.31–3.68)	0.003	1.61 (1.20–2.16)	0.002
Q5 (Highest)	0.90 (0.53–1.52)	0.691	2.66 (1.55–4.56)	< 0.001	2.80 (1.72–4.55)	< 0.001	1.94 (1.45–2.59)	< 0.001
Model 2.1 (Smoking variable only)								
Smoking status and frequency								
Non-smokers	Ref	Ref	Ref	Ref	Ref	Ref	Ref	Ref
Smokers—less than 1 per day	1.06 (0.48–2.34)	0.891	1.85 (1.00–3.41)	0.048	1.78 (0.96–3.30)	0.065	1.60 (1.10–2.32)	0.014
Smokers—1 to 5 per/day	0.81 (0.45–1.46)	0.491	1.06 (0.70–1.59)	0.776	1.63 (0.99–2.68)	0.055	1.14 (0.87–1.50)	0.342
Smokers—6 to 10 per day	1.39 (0.71–2.73)	0.332	1.22 (0.71–2.11)	0.465	1.35 (0.24–7.49)	0.731	1.28 (0.85–1.92)	0.234
Smokers—11 or more per day	2.12 (1.11–4.04)	0.022	0.90 (0.38–2.10)	0.803	2.55 (0.48–13.48)	0.271	1.52 (0.94–2.48)	0.090
Model 2.2 (Alcohol intake variable only)								
Alcohol intake status and frequency								
Non-drinkers	Ref	Ref	Ref	Ref	Ref	Ref	Ref	Ref
Drinkers—occasionally/not every week	1.33 (0.87–2.05)	0.186	1.35 (0.92–1.97)	0.119	1.42 (0.98–2.05)	0.062	1.35 (1.08–1.70)	0.008
Drinkers—once a week	0.87 (0.52–1.44)	0.583	2.11 (1.31–3.41)	0.002	1.56 (0.97–2.50)	0.065	1.45 (1.11–1.90)	0.007
Drinkers—2 to 3 times a week	1.02 (0.61–1.72)	0.936	1.52 (0.94–2.45)	0.087	1.93 (1.16–3.21)	0.011	1.45 (1.09–1.92)	0.011
Drinkers—everyday	0.94 (0.23–3.93)	0.938	6.11 (1.35–27.57)	0.019	6.83 (1.67–28.00)	0.008	2.79 (1.30–5.97)	0.008
Model 3.1 (Both SES and smoking were included in the same model)								
Household SES in quintiles								
Q1 (Lowest)	Ref	Ref	Ref	Ref	Ref	Ref	Ref	Ref
Q2 (Second lowest)	0.52 (0.28–0.97)	0.039	1.48 (0.96–2.30)	0.078	1.43 (0.89–2.30)	0.139	1.10 (0.83–1.46)	0.486
Q3 (Middle)	0.81 (0.52–1.26)	0.349	2.01 (1.22–3.32)	0.006	2.38 (1.44–3.91)	0.001	1.60 (1.22–2.09)	0.001
Q4 (Upper middle)	0.58 (0.30–1.12)	0.104	2.24 (1.42–3.54)	0.001	2.05 (1.22–3.47)	0.007	1.59 (1.18–2.13)	0.002
Q5 (Highest)	0.89 (0.52–1.51)	0.668	2.63 (1.52–4.55)	0.001	2.61 (1.59–4.29)	< 0.001	1.89 (1.41–2.54)	< 0.001
Smoking status and frequency								
Non-smokers	Ref	Ref	Ref	Ref	Ref	Ref	Ref	Ref
Smokers—less than 1 per day	1.04 (0.47–2.32)	0.921	1.75 (0.95–3.23)	0.074	1.59 (0.85–2.97)	0.144	1.55 (1.067–2.26)	0.022
Smokers—1 to 5 per day	0.80 (0.44–1.45)	0.460	0.97 (0.64–1.46)	0.890	1.36 (0.82–2.26)	0.236	1.05 (0.80–1.38)	0.743
Smokers—6 to 10 per day	1.37 (0.70–2.71)	0.360	1.10 (0.63–1.93)	0.723	1.01 (0.17–5.77)	0.994	1.21 (0.80–1.82)	0.363
Smokers—11 or more per day	2.13 (1.11–4.08)	0.022	0.81 (0.35–1.91)	0.637	2.08 (0.39–11.06)	0.391	1.45 (0.89–2.37)	0.135
Model 3.2 (Both SES and alcohol intake were included in the same model)								
Household SES in quintiles								
Q1 (Lowest)	Ref	Ref	Ref	Ref	Ref	Ref	Ref	Ref
Q2 (Second lowest)	0.51 (0.27–0.95)	0.034	1.46 (0.94–2.27)	0.087	1.35 (0.84–2.18)	0.217	1.07 (0.81–1.42)	0.618
Q3 (Middle)	0.81 (0.52–1.26)	0.344	1.99 (1.20–3.31)	0.007	2.27 (1.37–3.76)	0.002	1.53 (1.17–2.02)	0.002
Q4 (Upper middle)	0.56 (0.29–1.08)	0.083	2.18 (1.37–3.45)	0.001	2.02 (1.20–3.42)	0.009	1.54 (1.14–2.07)	0.005
Q5 (Highest)	0.89 (0.52–1.52)	0.673	2.44 (1.40–4.26)	0.002	2.40 (1.45–3.97)	0.001	1.78 (1.32–2.40)	< 0.001
Alcohol intake status and frequency								
Non-drinkers	Ref	Ref	Ref	Ref	Ref	Ref	Ref	Ref
Drinkers—occasionally/not every week	1.41 (0.91–2.18)	0.119	1.26 (0.86–1.84)	0.237	1.24 (0.85–1.80)	0.259	1.28 (1.02–1.60)	0.034
Drinkers—once a week	0.89 (0.53–1.49)	0.664	1.98 (1.23–3.21)	0.005	1.32 (0.82–2.13)	0.258	1.34 (1.02–1.76)	0.034
Drinkers—2 to 3 times a week	1.03 (0.60–1.76)	0.906	1.26 (0.78–2.05)	0.347	1.59 (0.94–2.68)	0.082	1.28 (0.96–1.70)	0.098
Drinkers—everyday	1.00 (0.24–4.18)	0.999	4.57 (0.99–21.04)	0.051	5.51 (1.32–22.97)	0.019	2.49 (1.16–5.35)	0.019
Model 4 (SES with both confounders (smoking and alcohol intake) in the same model)								
Household SES in quintiles								
Q1 (Lowest)	Ref	Ref	Ref	Ref	Ref	Ref	Ref	Ref
Q2 (Second lowest)	0.51 (0.27–0.94)	0.032	1.47 (0.94–2.28)	0.087	1.34 (0.83–2.16)	0.235	1.07 (0.81–1.42)	0.633
Q3 (Middle)	0.80 (0.51–1.26)	0.338	1.96 (1.18–3.26)	0.009	2.24 (1.35–3.72)	0.002	1.53 (1.17–2.01)	0.002
Q4 (Upper middle)	0.56 (0.29–1.09)	0.091	2.21 (1.40–3.51)	0.001	1.98 (1.17–3.36)	0.011	1.54 (1.14–2.07)	0.005
Q5 (Highest)	0.88 (0.51–1.52)	0.657	2.49 (1.42–4.34)	0.001	2.39 (1.44–3.97)	0.001	1.78 (1.32–2.41)	< 0.001
Smoking status and frequency								
Non-smokers	Ref	Ref	Ref	Ref	Ref	Ref	Ref	Ref
Smokers—less than 1 per day	1.05 (0.47–2.37)	0.904	1.60 (0.85–2.99)	0.143	1.48 (0.78–2.81)	0.229	1.44 (0.98–2.11)	0.062
Smokers—1 to 5 per/day	0.83 (0.45–1.53)	0.554	0.85 (0.56–1.31)	0.474	1.06 (0.60–1.86)	0.835	0.96 (0.72–1.28)	0.794
Smokers—6 to 10 per day	1.44 (0.72–2.88)	0.303	0.98 (0.55–1.74)	0.956	0.65 (0.11–3.84)	0.638	1.12 (0.73–1.70)	0.603
Smokers—11 or more per day	2.20 (1.13–4.25)	0.019	0.70 (0.30–1.65)	0.420	1.43 (0.25–8.17)	0.688	1.32 (0.80–2.17)	0.270
Alcohol intake status and frequency								
Non-drinkers	Ref	Ref	Ref	Ref	Ref	Ref	Ref	Ref
Drinkers—occasionally/not every week	1.39 (0.90–2.16)	0.139	1.24 (0.84–1.83)	0.267	1.18 (0.81–1.74)	0.386	1.24 (0.99–1.56)	0.065
Drinkers—once a week	0.86 (0.51–1.47)	0.592	2.03 (1.24–3.33)	0.005	1.27 (0.78–2.07)	0.333	1.31 (0.99–1.73)	0.058
Drinkers—2 to 3 times a week	1.00 (0.58–1.74)	0.984	1.29 (0.77–2.14)	0.330	1.55 (0.88–2.74)	0.132	1.24 (0.92–1.68)	0.161
Drinkers—everyday	0.81 (0.19–3.47)	0.776	4.75 (1.00–22.54)	0.050	5.41 (1.20–24.30)	0.028	2.32 (1.06–5.07)	0.034

Data are presented as odds ratio (OR) and 95% confidence interval (CI). All models were controlled for the potential effect of age, gender, and country (in the combined sample only).

Contrary to the above findings, South Africans with a household SES score in the middle and upper SES quintiles (Q3–Q5) were at higher risk of developing an NCD or multimorbidity compared to their lowest (Q1) SES counterparts (Table 2). Also, the regression analyses revealed that those who reported a smoking frequency of less than one per day and consumed alcohol once a week and everyday were more likely to be diagnosed with an NCD or multiple NCDs compared to their non-smoking or -drinking counterparts (models 2.1 and 2.2). Notably, the effects of smoking (Model 3.1) and alcohol intake (Model 3.2) had a small impact on the relationship between SES and NCD risk (Table 2).

Like SA, a one-unit increase in SES quintile group was associated with a higher risk of developing NCD in the Kenyan youth (Table 2). Specifically, Kenyans with a SES score in Q3, Q4, and Q5 had a twofold greater risk of developing NCDs compared to their lowest (Q1) SES counterparts. Smoking was not significantly associated with the risk of developing NCD in Kenya. However, respondents that consumed alcohol, especially those who drank “2–3 times a week” and “everyday” were at greater risk of developing NCD (Table 2). Adjusting for smoking and alcohol intake decreased the relative log odds of SES in increasing NCD risk. For instance, the risk of developing NCD in those in Q5 declined from 2.796 (1.719–4.549; p < 0.001)) (model 1) to 2.395 (1.442–3.974; p = 0.001) in model 4 after adjusting for both confounders (Table 2).

In the combined sample, those in the middle and upper (Q3, Q4, and Q5) SES groups had higher risk of developing an NCD or multimorbidity compared to their poorest (Q1) counterparts (Model 1). Models 2.1 and 2.2 revealed that those who reported smoking frequency of less than one per day and those who consumed alcohol were more likely to be diagnosed with an NCD(s) (Table 2). Subsequently, after adjusting for smoking and alcohol intake, the ORs of SES on the likelihood of being diagnosed with an NCD or NCD multimorbidity decreased (Table 2). For instance, the ORs of the highest (Q5) SES group decreased from 1.937 (1.448–2.589; p < 0.001) in model 1 to 1 1.785 (1.324–2.407; p < 0.001) in model 4 after adjusting for both moderators (Table 2).

## Discussion

Our main findings showed that the strength and direction of the SES–NCD association at different levels of SES varies within and between countries. In the UK, we showed that those in the second lowest SES quintile were less likely to develop NCDs than their lowest SES counterparts. In contrast, in SA and Kenya, respondents with SES scores between middle and highest quintiles were more likely to develop NCDs compared to the lowest SES quintile group. Furthermore, we reported a high incidence of smoking and alcohol use in all countries, with SA having the highest levels. Additionally, we showed that smoking and/or alcohol intake were associated with higher odds of developing NCDs in all countries. Subsequently, smoking and/or alcohol consumption had confounding effects on the SES–NCD relationships as evidenced by the decrease in the ORs of SES, especially in young adults from SA and Kenya. However, the SES–NCD associations remained strong and significant even after adjusting for smoking and/or alcohol intake. This implies that SES is a more prominent risk factor for developing NCDs when compared to smoking and/or alcohol intake, particularly in African countries such as SA and Kenya.

The findings that a higher SES score was associated with a higher risk of developing NCDs in SA and Kenya is in accordance with several reports from these two countries^18,19,25^, as well as, from other LMICs such as India and Indonesia^26,27^. However, they contradict those reported from Iran, in which a low SES was associated with NCD multimorbidity^28^. The inconsistent findings might be explained by differences in the number; type of NCDs; and methods used (i.e., self-reports, measured, or both) in the calculation of “NCD score” or NCD multimorbidity. Other confounding factors could include differences in age groups of respondents tested between the studies, 18–35-year-olds (in our study) vs 20–70-year-olds participants as in the Iranian study^28^. Nonetheless, the positive association between SES–NCD risk in LMICs seems to be common and has been attributed to the rapid urbanisation and epidemiological transition, and their effects on lifestyle factors^18,19,25^. This notion is also supported by the findings that NCDs such as obesity, diabetes, and hypertension are prevalent in affluent groups, especially in countries undergoing urbanisation and epidemiological transition including SA and Kenya^18–20,25^. Accordingly, we noted that the SES–NCD relationship was stronger in Kenya (a lower middle-income country in the early stages of transition) than in SA (an upper middle-income country that is farthest along the transition). This corroborates the findings that rapid urbanisation and epidemiological transition are accompanied by changes in SES and lifestyle factors, including diet, smoking, and alcohol intake^18,19,25^. For instance, high consumption of unhealthy diets has been reported in high SES individuals compared to their low SES counterparts^18,25^.

It has also been proposed that the positive association between SES and NCD risk in LMICs that we reported might be explained by the fact that those with high SES typically have higher education levels, thus have better health literacy and presumably more access to healthcare services^27^. Therefore, they are more likely to be diagnosed with NCDs earlier in life than their low SES counterparts. Indeed, studies conducted in SA and Kenya have shown that the distribution and the use of healthcare facilities, which include the majority of public and all private health sectors favour those with high SES^29,30^.

Our current findings in the UK sample corroborate other findings reported in the UK and other HICs^15,17,31,32^, in which lower SES groups have unfavourable health outcomes including a greater risk of developing NCDs (i.e., cancer and CVD) and dying prematurely compared to their high SES counterparts. The exact mechanisms underlying the health inequalities in the UK are complex and include many aspects such as social, economic, and environmental determinants^33^. These determinants, which are often interlinked, have also been reported as major drivers of health inequalities including unhealthy lifestyle behaviors^33^. Specifically, lower SES groups or more deprived areas are more likely to present with higher prevalence of risky behaviours and being admitted to hospital for alcohol related conditions compared to their counterparts^33^. Fortunately, the National Health Services has produced a 10-year plan, Long Term Plan, which includes many actions and priorities such as increasing funding to tackle social and health inequalities in vulnerable groups and communities^34^. Additionally, the Long Term Plan will also increase the development of prevention programmes to reduce smoking and obesity and increase participation in the established intervention programmes such as the Diabetes Prevention Programme^34^.

The findings that smoking and/or alcohol intake are associated with a higher risk of developing NCDs are in accordance with previous literature^11,12,22,35^. However, smoking and/or alcohol intake are declining in HICs yet are rapidly increasing in LMICs like SA and Kenya^11,12,22,35^. This may explain the alarming higher levels of smoking and alcohol use in SA and Kenya, when compared to the UK, more especially in SA. In SA and other African countries there are lower and weaker tobacco and alcohol polices in comparison to HICs^36,37^. Without aggressive policies and interventions targeting NCD risk factors, we foresee that populations in SA and Kenya will continue to suffer from the burden of NCDs.

It is important to point-out that in all three countries only a small proportion of respondents were presumably not diagnosed with an NCD condition. These findings are alarming and highlight the high burden of NCDs including in this young group of adults, which was previously rare. Specifically, our findings show that mental health conditions are still more common in HICs and that they are rapidly increasing in LMICs, where more than a quarter of respondents reported a diagnosis of a mental health problem. These findings are in accordance with previous reports^38,39^. We recently conducted a national survey with weighted data representing approximately 39.6 million South African households and showed that 25.7% and 17.8% of the South African population reported moderate to severe symptoms of probable depression and anxiety, respectively^39^. Furthermore, the findings that hypertension and related cardiovascular disorders (e.g., heart attack) was more prevalent in SA and Kenya than in the UK sample agrees with previous studies^40,41^. Specifically, the Sub-Saharan Africa region has the highest prevalence of hypertension in children (6.9%) and adults (27%)^40,41^. Additionally, a population-based cross-sectional study performed in four countries (Burkina Faso, Ghana, Kenya, and SA), showed that SA (47.6%) and Kenya (25.6%) had the highest levels of hypertension^5^. In SA, six out of the ten leading deaths in 2016 were NCDs and four of these diseases were hypertension (ranked 5th) and related disorders including “other forms of heart disease” (ranked 3rd), cerebrovascular disease (ranked 4th), and ischaemic heart disease (ranked 9th). These diseases accounted for 17.4% of mortality in SA in 2016^42^. In Kenya, it was reported that CVDs accounted for more than 12% of total deaths in 2016^43^.

Living with an NCD, especially with multiple NCDs has negative implications on the individual, household, and economy^26,27,44^. These include catastrophic health expenditure or out-of-pocket expenditure for the patient and their household due to long-term health care^26,27,44^. This may create a vicious cycle of poverty, especially if the patients and their household are already in poverty. NCDs are also associated with increased use of healthcare services as evidenced by high number of outpatient and inpatient visits in people with NCDs^27,29^. This poses a major threat to the healthcare systems, particularly in SA and Kenya, where there is still a high demand for healthcare services due to the existing burden of infectious diseases such as tuberculosis (TB), human immunodeficiency virus infection and acquired immunodeficiency syndrome (HIV/AIDS), and malaria^6,45^.

There is a need for a robust global multi-approach to reduce modifiable risk factors and subsequently lower the risk of NCD prevalence, particularly in LMICs. This can be achieved by implementing early preventative strategies to promote healthy lifestyle behaviours, increase knowledge and awareness about NCDs, and their associated risk factors. African countries, especially SA needs stricter and more innovative NCD risk factor policies than the current ones^37^, which will target smoking and alcohol intake behaviours in young adults. Such policies need to be multilevel and should consider broader societal issues such as lack of employment and recreational facilities that drive youth to engage in these unhealthy behaviours. With the increasing prevalence of NCDs and related deaths continuously overtaking infectious diseases^1–3,9^, local and international funding from the government and related organisations need to start prioritising NCDs over infectious diseases so that more resources are allocated in managing NCDs^7,8^. Additionally, primary healthcare and management clinical guidelines in LMICs need to reflect the trends in global health, thus need to shift from single disease (infectious disease) models and focus on multiple diseases or comorbidities to tackle the burden of NCDs^46–48^. Moreover, LMICs must urgently provide and increase access to mental health care services including counselling programmes for vulnerable groups and those already living with mental health conditions to avoid a mental health crisis in the near future.

This study was based on cross-sectional data which cannot show a causal relation between SES and NCD risk. Furthermore, we did not conduct medical tests such as an oral glucose tolerance test or measure blood pressure levels to confirm or diagnose NCD or NCD multimorbidity. Rather, we assumed that the respondents were aware of their NCD status after being told or diagnosed by a doctor or healthcare professional. Also, we would like to acknowledge that this approach has some limitations since there is a dearth of knowledge and awareness about NCDs and associated risk factors in LMICs. However, this is the only feasible method/approach for online surveys. Our study was limited to young adults, aged 18–35 years with internet access, which is not representative of the entire youth or general population. Therefore, our findings should be interpreted in relation to the respondents that were targeted. We would like to acknowledge that the household asset index is not the only measure of SES and that its use might be less appropriate for the UK sample. However, given that many respondents in our sample are from LMICs we decided to use the household assets index as a proxy of SES. Not only is the household assets index a preferred measure of SES in LMICs, it has also been shown to be central in the economic assessment of the household and sensitive to change over time^49^. Nonetheless, understanding the epidemiology of NCDs in young adults has the potential to decrease the risk of developing NCDs and related deaths via targeted interventions of NCD risk factors. Despite these limitations, our study used a large sample of participants from three countries with different levels of economic development, which enabled us to provide a detailed analysis of the impact of SES (in quintiles) and its association with NCD risk.

In conclusion, we showed that the strength and direction of SES–NCD associations differed within and between countries. Our findings highlight the urgent need for preparing healthcare systems and implementing tailored interventions and policies for addressing the burden of NCDs and associated risk factors, especially in LMICs.

## Supplementary Information

Supplementary Information 1.

Supplementary Information 2.

Supplementary Information 3.

## Data Availability

The datasets used and/or analysed during the current study are available from the corresponding author on reasonable request and will soon be available on the website (https://www.wits.ac.za/coe-human/research/).

## References

[1] NCD Countdown 2030 collaborators (2018). NCD Countdown 2030: Worldwide trends in non-communicable disease mortality and progress towards Sustainable Development Goal target 3.4. Lancet.

[2] World Health Organization. *Noncommunicable Diseases Country Profiles 2018* (2018).

[3] Gouda HN (2019). Burden of non-communicable diseases in sub-Saharan Africa, 1990–2017: Results from the Global Burden of Disease Study 2017. Lancet Glob. Health.

[4] Juma K, Juma PA, Shumba C, Otieno P, Asiki G (2020). Non-communicable diseases and urbanization in African Cities: A narrative review. Public Health in Developing Countries—Challenges and Opportunities.

[5] Gómez-Olivé FX (2017). Regional and sex differences in the prevalence and awareness of hypertension: An H3Africa AWI-Gen study across 6 sites in sub-Saharan Africa. Glob. Heart.

[6] World Health Organization. *WHO Global Lists of High Burden Countries for Tuberculosis (TB), TB/HIV and Multidrug/Rifampicin-Resistant TB (MDR/RR-TB), 2021–2025*. http://apps.who.int/bookorders (2021).

[7] Lemoine M, Girard PM, Thursz M, Raguin G (2012). In the shadow of HIV/AIDS: Forgotten diseases in sub-Saharan Africa: Global health issues and funding agency responsibilities. J. Public Health Policy.

[8] National Academies of Sciences, Engineering & Medicinde. *Global Health and the Future Role of the United States* (2017). 29001490

[9] Murray CJL (2020). Global burden of 87 risk factors in 204 countries and territories, 1990–2019: A systematic analysis for the Global Burden of Disease Study 2019. Lancet.

[10] World Health Organization. Noncommunicable Diseases. https://www.who.int/news-room/fact-sheets/detail/noncommunicable-diseases (2021).

[11] World Health Organization. *WHO Global Report on Trends in Prevalence of Tobacco Use 2000–2025, fourth edition*. http://apps.who.int/bookorders (2021).

[12] World Health Organization. *Global Status Report on Alcohol and Health 2018* (2018).

[13] Stringhini S (2017). Socioeconomic status and the 25 × 25 risk factors as determinants of premature mortality: A multicohort study and meta-analysis of 1·7 million men and women. Lancet.

[14] GBD 2019 Viewpoint Collaborators (2020). Five insights from the Global Burden of Disease Study 2019. Lancet.

[15] Kyrou I (2020). Sociodemographic and lifestyle-related risk factors for identifying vulnerable groups for type 2 diabetes: A narrative review with emphasis on data from Europe. BMC Endocr. Disord..

[16] McLaren L (2007). Socioeconomic status and obesity. Epidemiol. Rev..

[17] Roskam AJR (2010). Comparative appraisal of educational inequalities in overweight and obesity among adults in 19 European countries. Int. J. Epidemiol..

[18] Steyn NP, Parker W, Nel JH, Ayah R, Mbithe D (2011). Dietary, social, and environmental determinants of obesity in Kenyan women. Scand. J. Public Health.

[19] Nienaber-Rousseau C (2017). Socio-demographic and lifestyle factors predict 5-year changes in adiposity among a Group of Black South African Adults. Int. J. Environ. Res. Public Health.

[20] Hanna DR, Campbell JA, Walker RJ, Dawson AZ, Egede LE (2021). Association between health and wealth among Kenyan adults with hypertension. Glob. J. Health Sci..

[21] van Niekerk, W. & le Roux, A. Chapter 10: Human Settlements. in *Climate Risk and Vulnerability: A Handbook for Southern Africa* (2017).

[22] Boua PR (2021). Prevalence and socio-demographic correlates of tobacco and alcohol use in four sub-Saharan African countries: A cross-sectional study of middle-aged adults. BMC Public Health.

[23] Nonterah EA (2021). Poor cardiovascular health is associated with subclinical atherosclerosis in apparently healthy sub-Saharan African populations: An H3Africa AWI-Gen study. BMC Med..

[24] Ramsay M (2018). Regional and sex-specific variation in BMI distribution in four sub-Saharan African countries: The H3Africa AWI-Gen study. Glob. Health Action.

[25] Mwangi KJ (2020). Socio-economic and demographic determinants of non-communicable diseases in Kenya: A secondary analysis of the Kenya stepwise survey. Pan Afr. Med. J..

[26] Reddy MM (2022). Prevalence, associated factors, and health expenditures of noncommunicable disease multimorbidity—findings from gorakhpur health and demographic surveillance system. Front. Public Health.

[27] Marthias T (2021). Impact of non-communicable disease multimorbidity on health service use, catastrophic health expenditure and productivity loss in Indonesia: A population-based panel data analysis study. BMJ Open.

[28] Khorrami Z (2020). The patterns of non-communicable disease multimorbidity in Iran: A multilevel analysis. Sci. Rep..

[29] Oyando R, Barasa E, Ataguba JE (2022). Socioeconomic inequity in the screening and treatment of hypertension in Kenya: Evidence From a National Survey. Front. Health Serv..

[30] Ataguba JE, Akazili J, Mcintyre D (2011). Socioeconomic-related health inequality in South Africa: Evidence from General Household Surveys. Int. J. Equity Health.

[31] Exarchakou A, Kipourou DK, Belot A, Rachet B (2022). Socio-economic inequalities in cancer survival: How do they translate into Number of Life-Years Lost?. Br. J. Cancer.

[32] Corris V (2020). Health inequalities are worsening in the North East of England. Br. Med. Bull..

[33] Public Health England. Chapter 5: Inequalities in health. https://www.gov.uk/government/publications/health-profile-for-england-2018 (2018).

[34] National Health Service. NHS Long Term: Overview and Summary. https://www.longtermplan.nhs.uk/online-version/overview-and-summary/.

[35] Kendagor A (2018). Prevalence and determinants of heavy episodic drinking among adults in Kenya: Analysis of the STEPwise survey, 2015. BMC Public Health.

[36] Nishio A (2018). Systematic review of school tobacco prevention programs in African countries from 2000 to 2016. PLoS One.

[37] Ndinda C, Ndhlovu TP, Juma P, Asiki G, Kyobutungi C (2018). The evolution of non-communicable diseases policies in post-apartheid South Africa. BMC Public Health.

[38] Kazdin AE (2021). Antidepressant use in low-middle- and high-income countries: A World Mental Health Surveys report. Psychol. Med..

[39] Craig A (2022). The prevalence of probable depression and probable anxiety, and associations with adverse childhood experiences and socio-demographics: A national survey in South Africa. Front. Public Health.

[40] World Health Organization. Hypertension. https://www.who.int/news-room/fact-sheets/detail/hypertension (2021).

[41] Song P (2019). Global prevalence of hypertension in children: A systematic review and meta-analysis. JAMA Pediatr..

[42] Statistics South Africa (2018). Mortality and Causes of Death in South Africa, 2016: Findings from Death Notification.

[43] Asiki G (2018). Policy environment for prevention, control and management of cardiovascular diseases in primary health care in Kenya. BMC Health Serv. Res..

[44] Verma VR, Kumar P, Dash U (2021). Assessing the household economic burden of non-communicable diseases in India: Evidence from repeated cross-sectional surveys. BMC Public Health.

[45] Kalonji D, Mahomed OH (2019). Health system challenges affecting HIV and tuberculosis integration at primary healthcare clinics in Durban, South Africa. Afr. J. Prim. Health Care Fam. Med..

[46] UNAIDS. *Chronic care of HIV and Noncommunicable Diseases: How to Leverage the HIV Experience* (2011).

[47] Leung C (2016). Preparedness of HIV care and treatment clinics for the management of concomitant non-communicable diseases: A cross-sectional survey. BMC Public Health.

[48] Young F, Critchley JA, Johnstone LK, Unwin NC (2009). A review of co-morbidity between infectious and chronic disease in Sub Saharan Africa: TB and Diabetes Mellitus, HIV and Metabolic Syndrome, and the impact of globalization. Glob. Health.

[49] Kabudula CW (2017). Assessing changes in household socioeconomic status in Rural South Africa, 2001–2013: A distributional analysis using household asset indicators. Soc. Indic. Res..

