# Supplementary material for: Association between socio-economic status and non-communicable disease risk in young adults from Kenya, South Africa, and the United Kingdom
Source: Sci Rep. 2023 Jan 13;13:728. doi: 10.1038/s41598-023-28013-4 (PMC9839722; doi:10.1038/s41598-023-28013-4)
Supplement: Supplementary file 1 — Supplementary Information 1. [file 41598_2023_28013_MOESM1_ESM.pdf]

Title: The association of socio-economic status with non-communicable disease risk in young adults from Kenya, South Africa, and the United Kingdom

Supporting information

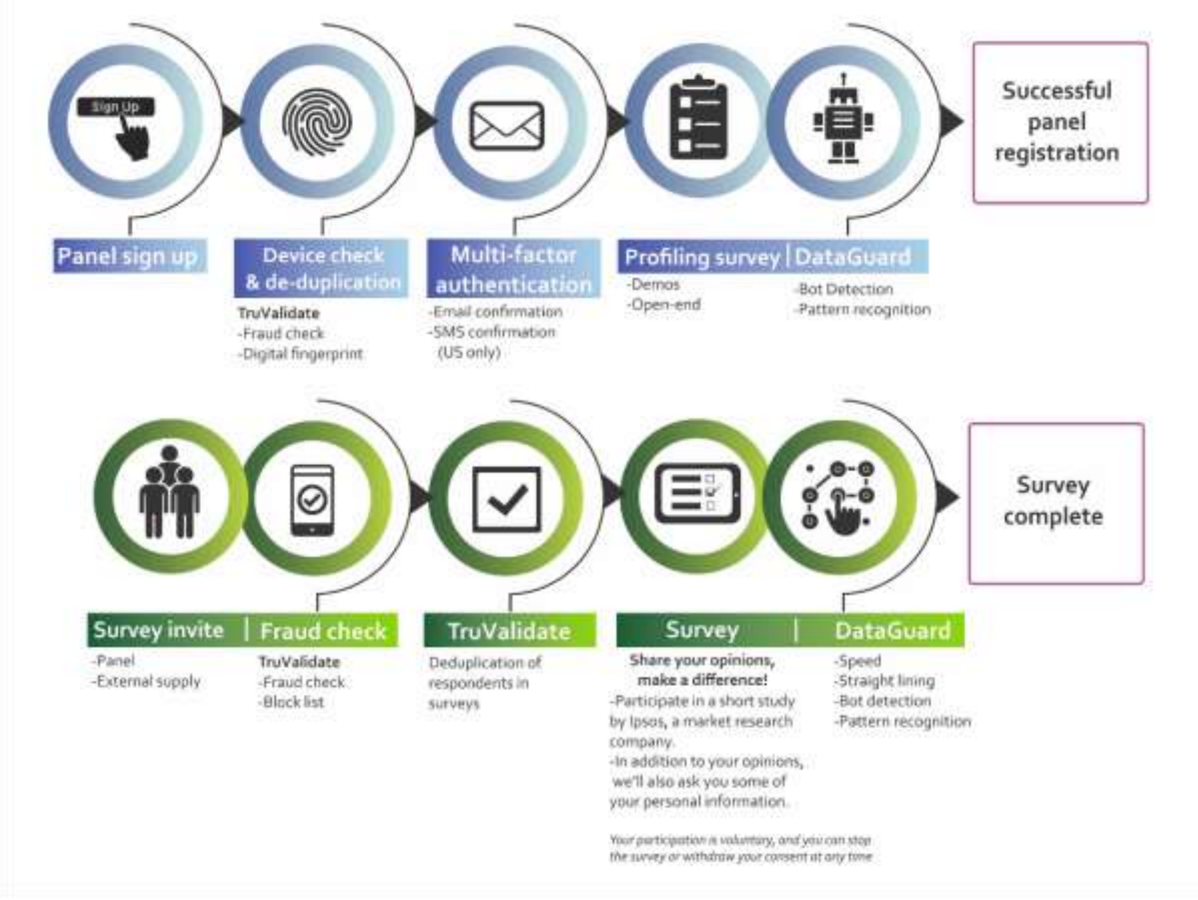

Figure S1. The international market research and public opinion polling company (IPSOS) "i-Say panel" used for the recruitment of study respondents.
