# Supplementary figures and images for: Association between socio-economic status and non-communicable disease risk in young adults from Kenya, South Africa, and the United Kingdom
Source: Sci Rep. 2023 Jan 13;13:728. doi: 10.1038/s41598-023-28013-4 (PMC9839722; doi:10.1038/s41598-023-28013-4)

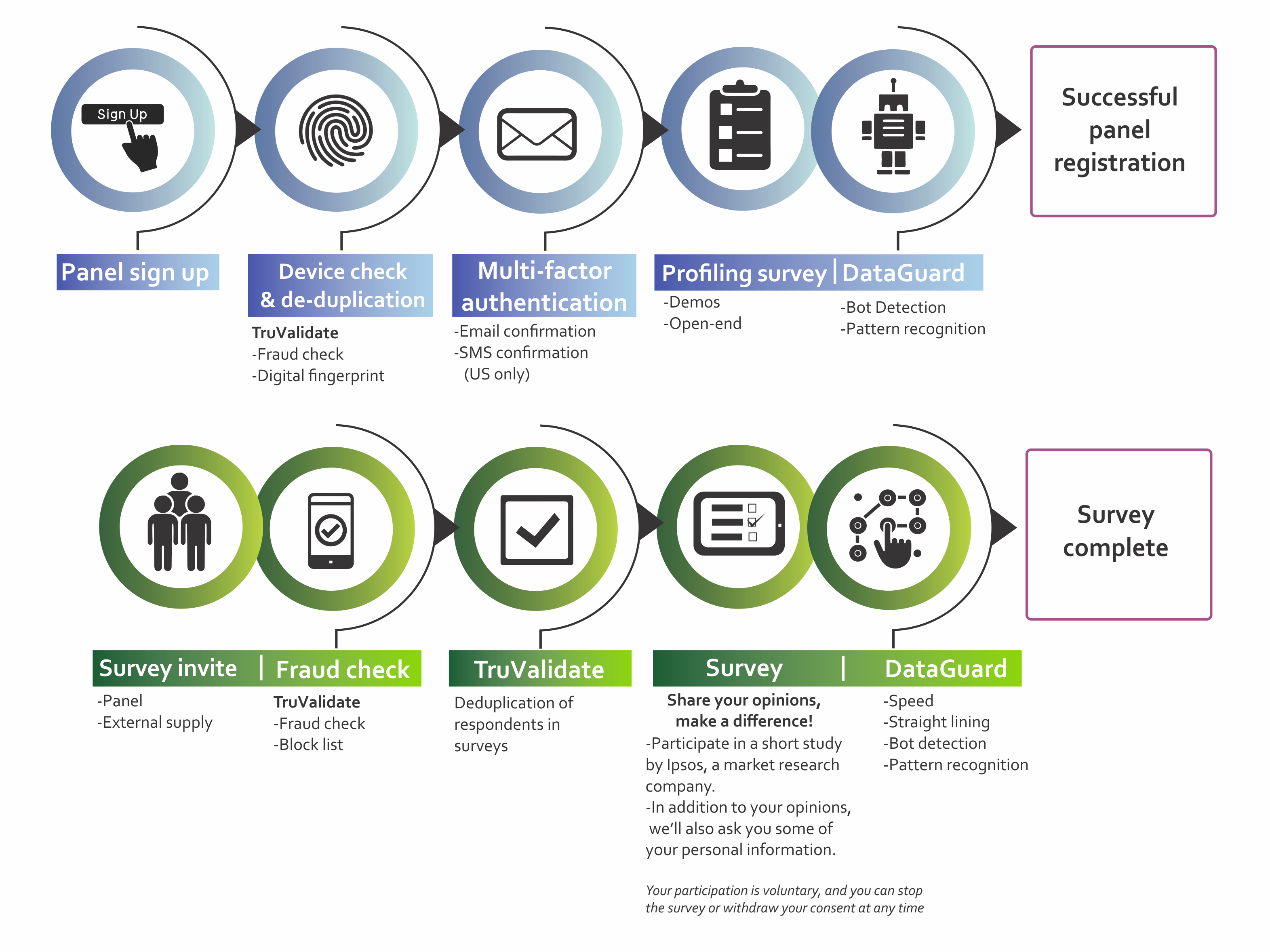

Supplement: Supplementary file 2 — Supplementary Information 2. [file 41598_2023_28013_MOESM2_ESM.png]
