# Supplementary material for: Association between socio-economic status and non-communicable disease risk in young adults from Kenya, South Africa, and the United Kingdom
Source: Sci Rep. 2023 Jan 13;13:728. doi: 10.1038/s41598-023-28013-4 (PMC9839722; doi:10.1038/s41598-023-28013-4)
Supplement: Supplementary file 3 — Supplementary Information 3. [file 41598_2023_28013_MOESM3_ESM.docx]

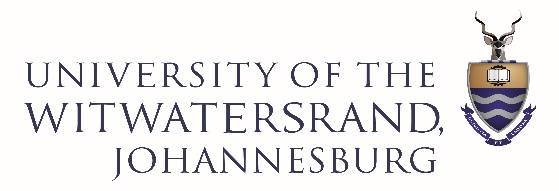

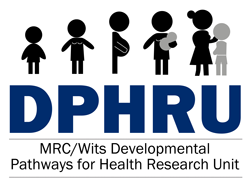

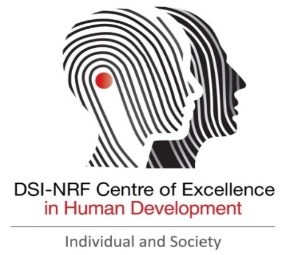


**Survey Questionnaire**

| **Section 1: Demographic and socio-economic characteristics** | | |
| --- | --- | --- |
| 1 | How old are you? | _________[number] |
| 2 | Marital status | 1 – Single  2 – Married/living together  3 – Separated  4 – Divorced  5 – Widowed |
| 3 | Which of the following are present in your household? **(Click for YES)** | |
| 3.1 | Furniture (Sofa or armchairs) |  |
| 3.2 | Mattress |  |
| 3.3 | Bed |  |
| 3.4 | Gas (or kerosene) cooker |  |
| 3.5 | Stove (electric or gas) |  |
| 3.6 | Refrigerator |  |
| 3.7 | Air conditioner |  |
| 3.8 | Washing machine |  |
| 3.9 | Bicycle |  |
| 3.10 | Motorbike |  |
| 3.11 | Cars and other 4-wheel vehicles |  |
| 3.12 | Generator |  |
| 3.13 | Fan |  |
| 3.14 | Microwave |  |
| 3.15 | Television |  |
| 3.16 | Computer or tablet |  |
| 3.17 | Satellite dish |  |
| 3.18 | Smartphone |  |
| 3.19 | Mobile phone (not smartphones) |  |
| 3.20 | Flush toilet in / outside house |  |
| 3.21 | Tap water in house/on plot |  |
| 3.22 | Electricity |  |
| 4 | What type of dwelling does the household live in? **(Click the one that best describes it)?** | |
| 4.1 | Separated house (Bungalow) |  |
| 4.2 | Semi-detached house/Cluster house/Townhouse |  |
| 4.3 | Flat/Apartment |  |
| 4.4 | Compound house (separate rooms, sharing facilities) |  |
| 4.5 | Huts/Buildings (shared compound) |  |
| 4.6 | Huts/Buildings (private compound) |  |
| 4.7 | Tents |  |
| 4.8 | Improvised home (kiosk, container, shack) |  |
| 4.9 | Living quarters attached to office/shop |  |
| 4.10 | Uncompleted building |  |
| 4.11 | Hostel |  |
| 4.12 | Room in backyard |  |
| 4.13 | Caravan |  |
| 5 | How many people, in total, who live in your household? When I say household, I mean all the people, including yourself, who live in your home and eat out of the same kitchen. **(Please enter the TOTAL number)**  *Please only include people who are currently living in your household, not people who are living away for long periods like weeks or months. Please include employees if they eat from the same kitchen and eat out of the same kitchen.* | _________[number] |
| 6 | How many people, in total, in each of these age categories, live in your household?  **(Click for YES for the appropriate number)** | |
| 6.1 | Children, aged 14 years and younger | 0 – No children  1 – 1 child  2 – 2 children  3 – 3 children  4 – 4 children  5 – 5 children  6 – More than 5 children |
| 6.2 | Adults, aged 15 – 17 | 0 – No adults  1 – 1 adult  2 – 2 adults  3 – 3 adults  4 – 4 adults  5 – 5 adults  6 – More than 5 adults |
| 6.3 | Adults, aged 18-24 | 0 – No adults  1 – 1 adult  2 – 2 adults  3 – 3 adults  4 – 4 adults  5 – 5 adults  6 – More than 5 adults |
| 6.4 | Adults, aged 25-34 | 0 – No adults  1 – 1 adult  2 – 2 adults  3 – 3 adults  4 – 4 adults  5 – 5 adults  6 – More than 5 adults |
| 6.5 | Adults, aged 35-49 | 0 – No adults  1 – 1 adult  2 – 2 adults  3 – 3 adults  4 – 4 adults  5 – 5 adults  6 – More than 5 adults |
| 6.6 | Adults, aged 50-64 | 0 – No adults  1 – 1 adult  2 – 2 adults  3 – 3 adults  4 – 4 adults  5 – 5 adults  6 – More than 5 adults |
| 6.7 | Adults, aged 65 or older | 0 – No adults  1 – 1 adult  2 – 2 adults  3 – 3 adults  4 – 4 adults  5 – 5 adults  6 – More than 5 adults |
| 7 | How many rooms are in your house (excluding bathrooms, store-rooms or garages)? | _________[number] |

| **Section 2: Co-morbidities and accessibility to health care services** | | |
| --- | --- | --- |
| 8 | Has a doctor or nurse or health worker at a clinic or at hospital told you that you had or have any of the following conditions **(Click for YES)**: | |
| 8.1 | Heart attack or angina (chest pains) |  |
| 8.2 | Stroke |  |
| 8.3 | High blood cholesterol or fats in the blood |  |
| 8.4 | Diabetes or blood Sugar |  |
| 8.5 | Overweight or obesity |  |
| 8.6 | HIV/AIDS |  |
| 8.7 | Asthma and chronic obstructive pulmonary disease (COPD) |  |
| 8.8 | Sore joints or muscle problems, e.g., Arthritis or gout |  |
| 8.9 | Tuberculosis (TB) |  |
| 8.10 | Cancer |  |
| 8.11 | Mental health condition (e.g., Depression, anxiety, bi-polar) |  |
| 8.12 | Liver disease |  |
| 8.13 | Chronic kidney disease |  |
| 8.14 | Hypertension or high Blood Pressure |  |
| **Accessibility to health care services** | | |
| 9 | In the past three months were you sick? | 0 – No  1 – Yes |
| 10 | If Yes, did you see a doctor or nurse or health worker at a clinic or at hospital? | 0 – No  1 – Yes |
| 11 | If No, what were the reasons for not seeking medical assistance? | 1 – Health care too far  2 – Shortage of health care workers (e.g., doctors, nurse)  3- Other (specify) |

| **Section 3: Blood pressure and lifestyle risk factors** | | |
| --- | --- | --- |
| 12 | Do you know what the side-effects are of having uncontrolled high blood pressure? | 0 – No  1 – Yes |
| 13 | How often do you get your blood pressure checked? | 1 – Once a month  2 – Once every three months  3 – Twice a year  4 – Once a year  5 – Never |
| 14 | If never, is this a result of the following: | 1 – Lack of transport to a health facility  2 – Your health care facility does not offer this procedure  3 – BP checking is not important for you  4- Other (specify) |
| 15 | If you have hypertension (high blood pressure), how long ago were you diagnosed? (**Choose the most appropriate response**) | 1– Less than a year ago  2 – More than a year ago  3 – More than 5 years ago  4 – More than 10 years ago |
| 16 | If you have hypertension (high blood pressure), are you currently on medication or undergoing treatment? | 0 – No  1 – Yes |
| **Lifestyle behavioural risk factors** | | |
| 17 | Do you smoke? | 0 – No  1 – Yes |
| 18 | If yes, how many do you smoke a day?  *This may include but not limited to cigarettes with a filter or rolled, chewing tobacco, pipe, cigars, vape, hubbly, snuff or dagga.* | 1 – Less than 1 per day  2 – 1 to 5 per day  3 – 6 to10 per day  4 – 11 to 20 per day  5 – More than 20 days |
| 19 | Do you drink alcohol? | 0 – No  1 – Yes |
| 20 | If yes, how many times during the week do you drink alcohol? | 1 – Occasionally / not every week  2 – Once a week  3 – 2 to 3 times a week  4 – Every day |

| **Section 4: General Nutrition Knowledge** | | |
| --- | --- | --- |
| This section is a survey and not a test. Your answers will help identify which dietary advice people find confusing. It is important that you complete it by yourself. If you do not know the answer, please mark “Not sure” rather than guess. | | |
| 21 | Do health experts recommend that people should be eating more, the same amount, or less of the following foods? **(Click one option per food)** | |
| 21.1 | Fruit | 1 –More  2 – Same  3 – Less  4 – Not sure |
| 21.2 | Food and drinks with added sugar | 1 –More  2 – Same  3 – Less  4 – Not sure |
| 21.3 | Vegetables | 1 –More  2 – Same  3 – Less  4 – Not sure |
| 21.4 | Fatty foods | 1 –More  2 – Same  3 – Less  4 – Not sure |
| 21.5 | Processed red meat | 1 –More  2 – Same  3 – Less  4 – Not sure |
| 21.6 | Whole grains | 1 –More  2 – Same  3 – Less  4 – Not sure |
| 21.7 | Salty foods | 1 –More  2 – Same  3 – Less  4 – Not sure |
| 21.8 | Water | 1 –More  2 – Same  3 – Less  4 – Not sure |
| 22 | How many servings of fruit and vegetables per day do experts advise people to eat as a minimum? (One serving could be, for example, an apple or a handful of chopped carrots) **(Click one)** | 1 – Two servings  2 – Three servings  3 – Four servings  4 – Five or more servings  5 – Not sure |
| 23 | Which of these types of fats do experts recommend that people should eat less of? (**Click one option per food)** | |
| 23.1 | Unsaturated fats | 1 – Eat less  2 – Not eat less  3 – Not sure |
| 23.2 | Trans fats | 1 – Eat less  2 – Not eat less  3 – Not sure |
| 23.3 | Saturated fats | 1 – Eat less  2 – Not eat less  3 – Not sure |
| 24 | Which type of dairy foods do experts say people should drink? **(Click one)** | 1 – Full fat (e.g., full fat milk)  2 – Reduced fat (e.g., skimmed and semi-skimmed milk)  3 – Mixture of full fat and reduced fat  4 – Neither dairy foods should be avoided  5 – Not sure |
| 25 | How many times per week do experts recommend that people eat oily fish (e.g., salmon and mackerel)? **(Click one)** | 1 – 1 to 2 times per week  2 – 3 to 4 times per week  3 – Every day  4 – Not sure |
| 26 | Approximately how many alcoholic drinks is the maximum recommended per day (The exact number depends on the size and strength of the drink)? **(Click one)** | 1 – 1 drink each for men and women  2 – 2 drinks each for men and women  3 – 2 drinks for men and 1 drink for women  4 – 3 drinks for men and 2 drinks for women  5 – Not sure |
| 27 | How many times per week do experts recommend that people eat breakfast? **(Click one)** | 1 –3 times per week  2 –4 times per week  3 – Every day  4 – Not sure |
| 28 | If a person has two glasses of fruit juice in a day, how many of their daily fruit and vegetable servings would this count as? **(Click one)** | 1 – None  2 – One serving  3 – Two servings  4 – Three servings  5 – Not sure |
| 29 | According to the ‘eatwell guide’ (a guideline showing the proportions of food types people should eat to have a balanced and healthy diet), how much of a person’s diet should be made up of starchy foods? **(Click one)** | 1 –Quarter  2 –Third  3 – Half  4 – Not sure |
| 30 | Do you have any nutrition related qualifications (or are you studying to get a nutrition qualification)? If yes, please specify. | _____________________ |

| **Section 5: Perceptions and understanding of obesity** | | |
| --- | --- | --- |
| We are interested in your beliefs/perceptions about other people’s weight, your own weight, and your opinions about policies designed to tackle obesity. | | |
| Please look at the two sets of ten pictures **(A-J)** of men and women in swimwear with increasing body mass index (BMI) and answer the relevant questions. | | |
| 31 | At what point, if at all, do you think the pictures show a man who is very overweight, sometimes referred to by doctors as 'obese'? Please just tell me the letter.  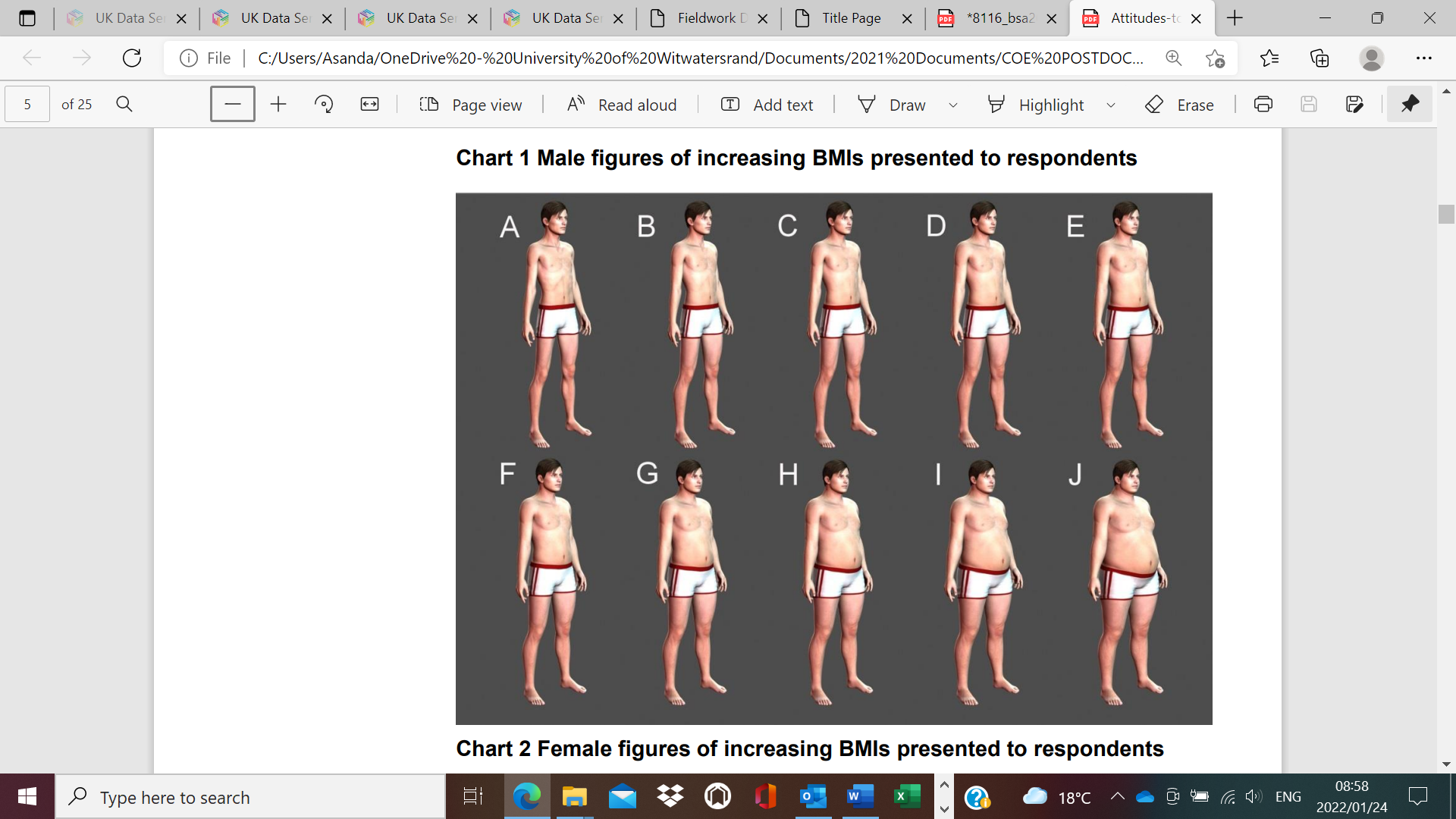 | 1 – A  2 – B  3 – C  4 – D  5 – E  6 – F  7 – G  8 – H  9 – I  10 – J  11 – None of these show a man who is obese |
| 32 | At what point, if at all, do you think the pictures show a woman who is very overweight, sometimes referred to by doctors as 'obese'? Please just tell me the letter.  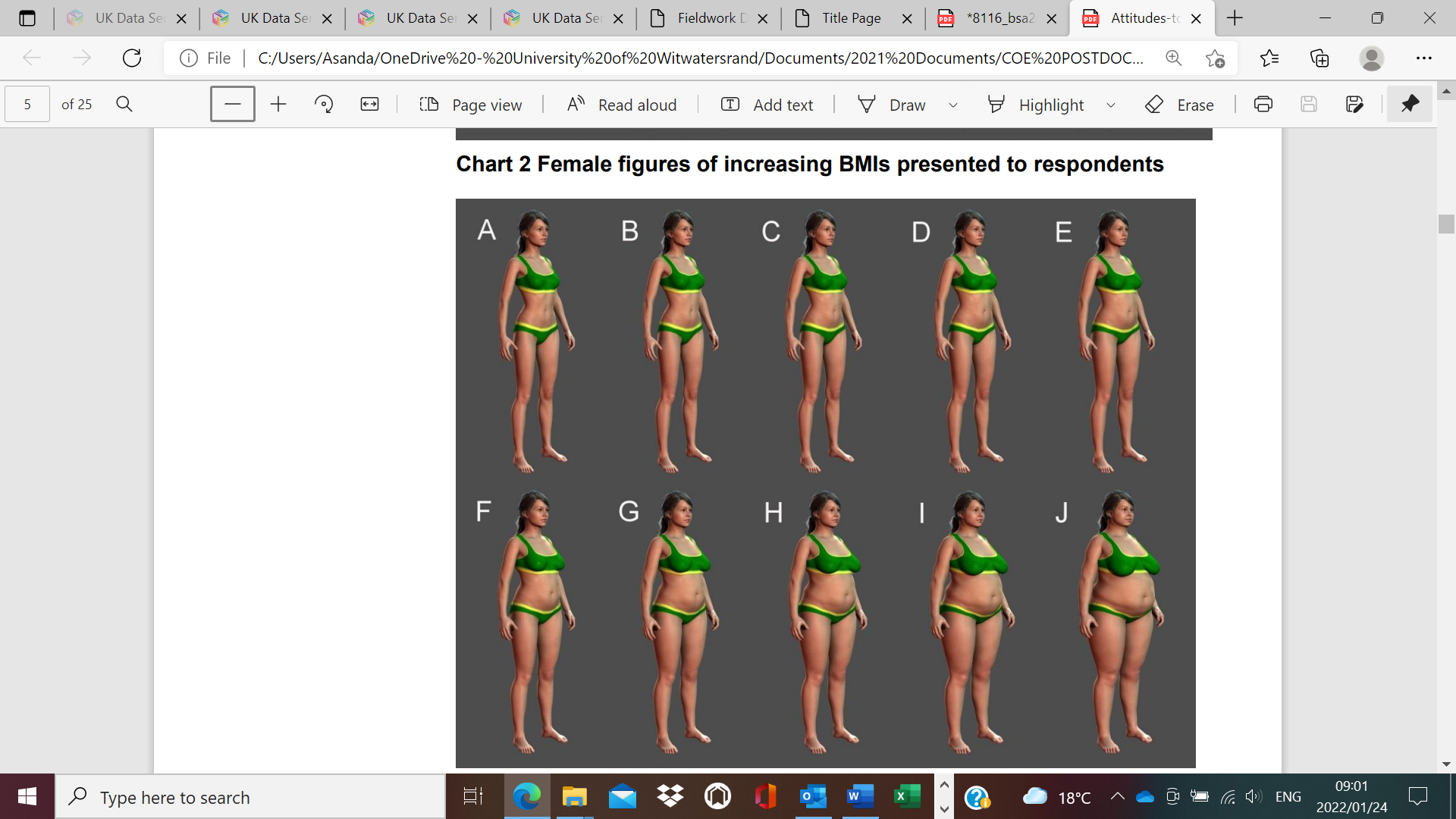 | 1 – A  2 – B  3 – C  4 – D  5 – E  6 – F  7 – G  8 – H  9 – I  10 – J  11 – None of these show a woman who is obese |
| 33 | We would like to ask you how you see your own body shape. Please look at this showcard again and tell me which of these pictures comes closest to what you think of as your own body shape? Please just tell me the letter **(Use male pictures for men; female pictures for women**).  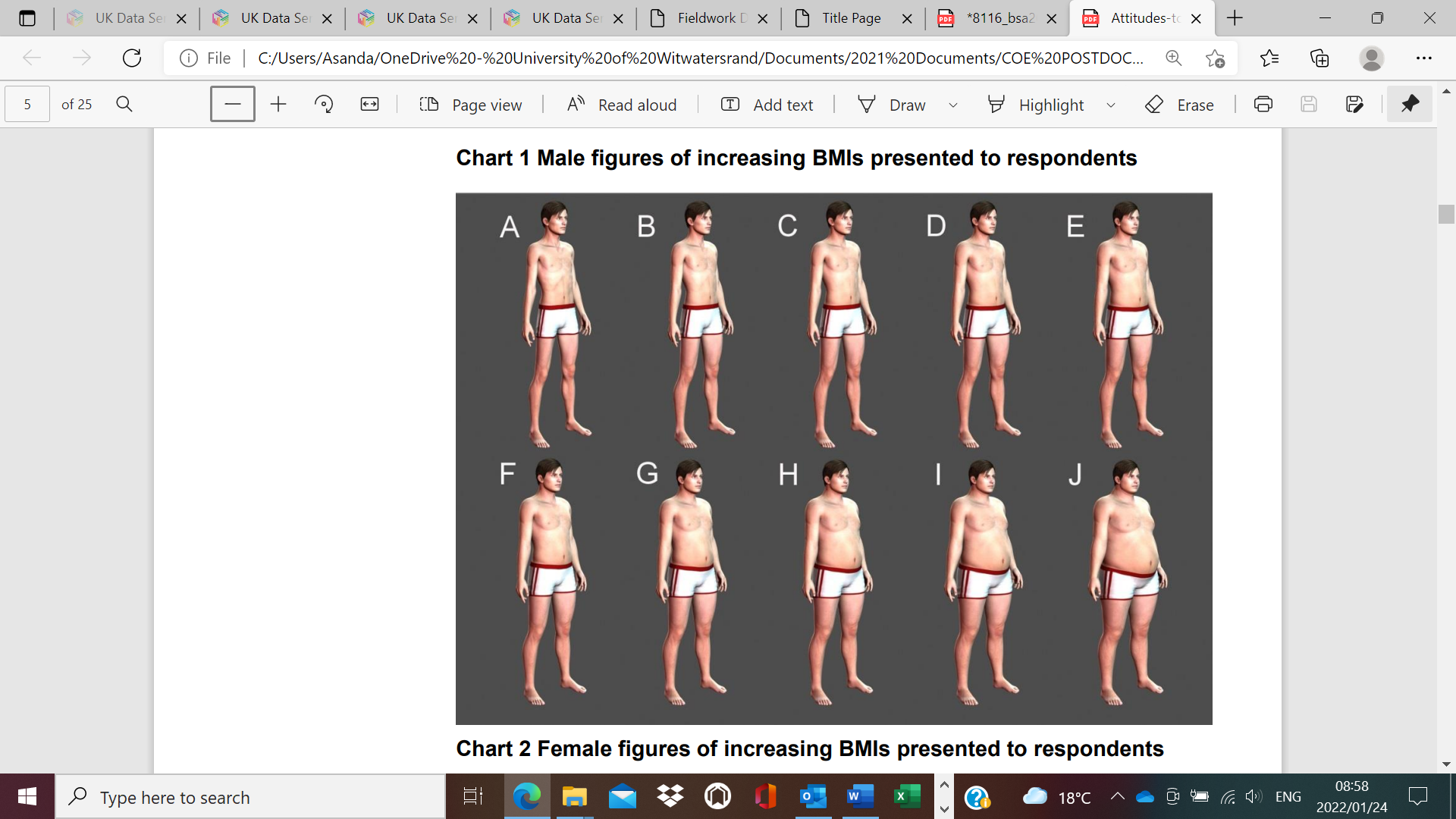  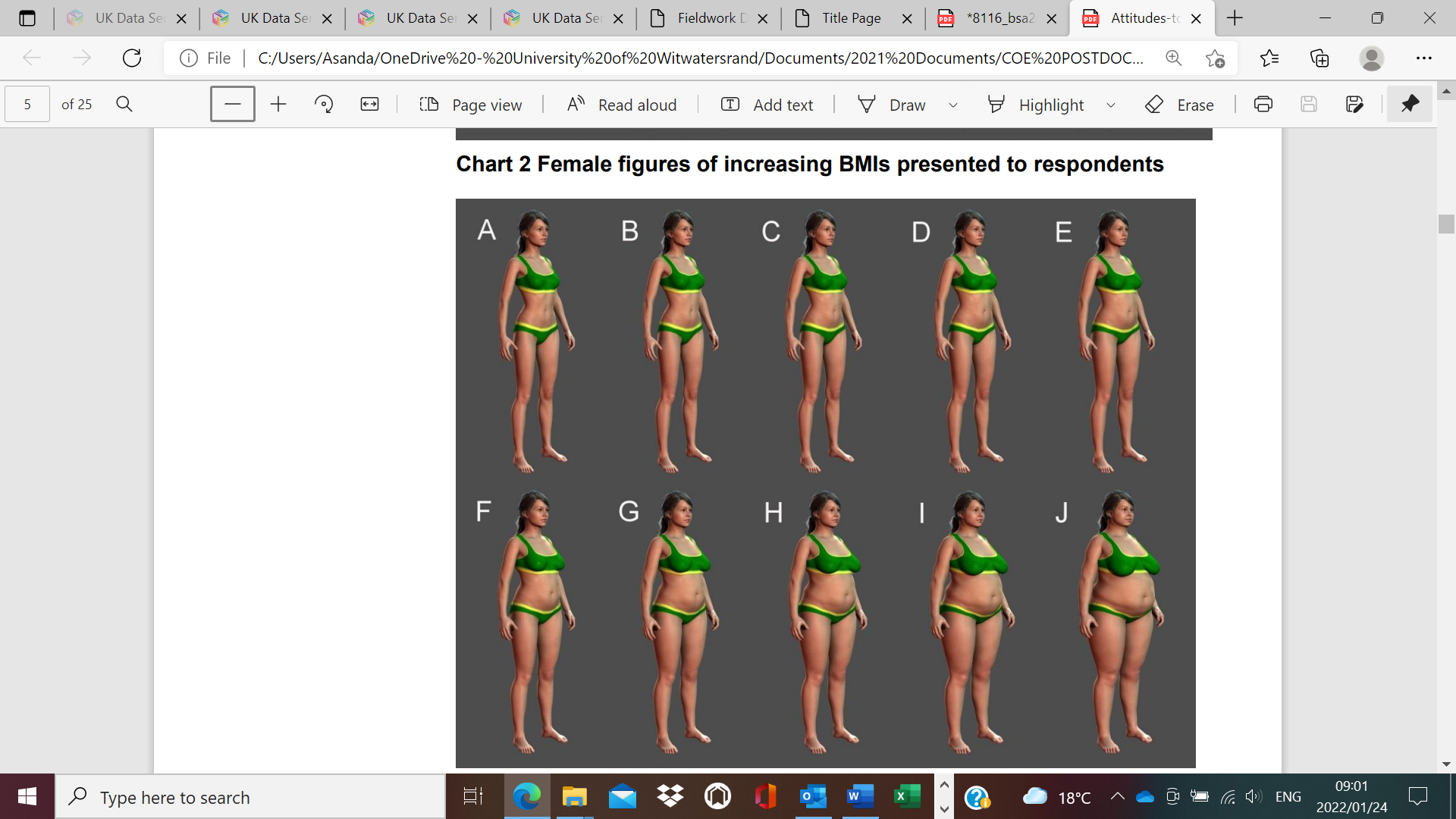 | 1 – A  2 – B  3 – C  4 – D  5 – E  6 – F  7 – G  8 – H  9 – I  10 – J  11 – None of these show a man/woman who is obese |
| 34 | Do you think of yourself as underweight? | 0 – No  1 – Yes  2 –Don't know |
| 35 | Do you think of yourself as about the right weight? | 0 – No  1 – Yes  2 –Don't know |
| 36 | Do you think of yourself as overweight? | 0 – No  1 – Yes  2 –Don't know |
| 37 | Are you happy with your own weight? | 1 – Happy  2–Neither happy nor unhappy  3– Unhappy |
| 38 | Please say how much you agree or disagree with the following **statements**: | |
| 38.1 | ‘There is no reason to worry about being a bit overweight’? | 1– Strongly agree  2 –Agree  3 – Neutral  4 –Disagree  5 –Strongly disagree |
| 38.2 | ‘Being overweight is something you inherit from your parents’?  *By 'inherit', we mean it is something your parents pass to you through their genes.* | 1– Strongly agree  2 –Agree  3 – Neutral  4 –Disagree  5 –Strongly disagree |
| 38.3 | ‘Most overweight people have put on weight because of low metabolism’?  *'Low metabolism' means your body burns calories more slowly than other people.* | 1– Strongly agree  2 –Agree  3 – Neutral  4 –Disagree  5 –Strongly disagree |
| 38.4 | 'Most people who are overweight have put on weight because they **eat** too much' | 1– Strongly agree  2 –Agree  3 – Neutral  4 –Disagree  5 –Strongly disagree |
| 38.5 | 'Most people who are overweight have put on weight because they **exercise** too little' | 1– Strongly agree  2 –Agree  3 – Neutral  4 –Disagree  5 –Strongly disagree |
| 38.6 | Here is a list of health conditions.  Which, if any, do you think a person is more likely to get if they are very overweight, sometimes referred to by doctors as 'obese'? | 1 – Arthritis (pain/swelling in the joints)  2 – Liver disease  3 – Asthma  4 –Depression  5 – Shingles (painful rash)  6 – Stroke  7 – High blood pressure/hypertension  8 –Type 2 diabetes/Blood sugar  9 –Some cancers  10 – Heart disease  11 –All of these  12 – None of these |

| **Section 6: Public opinion about obesity policies** | | |
| --- | --- | --- |
| 39 | Which, if any, of the people on this list do you think should be responsible for trying to reduce the number of people in your country who are very overweight, sometimes referred to by doctors as 'obese'? | 1 – Gyms or local leisure centres  2 – Companies that help people diet (e.g., WeightWatchers)  3 – The media  4 – Supermarkets  5 – Food and drink manufacturers  6 – Health care professionals (e.g., doctors or nurses)  7 – The government  8 – Family and friends of people who are very overweight (obese)  9 – Individuals who are very overweight (obese) themselves  10 – All of these  11 – None of these |
| 40 | Below are some things that some people have suggested might help reduce the number of people in your country who are very overweight. For each one, please say whether you are in **favour or against** this idea. | |
| 40.1 | Putting a tax on high fat foods, which would increase the price of things like crisps and chocolate | 1 – Strongly in favour  2 – In favour  3 – Neutral  4 – Against  5 – Strongly against |
| 40.2 | Putting a tax on sugary fizzy drinks - are you in favour or against this idea?  *Note - 'sugary fizzy drinks' does not include fruit juice.* | 1 – Strongly in favour  2 – In favour  3 – Neutral  4 – Against  5 – Strongly against |
| 40.3 | Banning adverts for high fat foods, like crisps and chocolate? | 1 – Strongly in favour  2 – In favour  3 – Neutral  4 – Against  5 – Strongly against |
| 40.4 | Banning adverts for sugary fizzy drinks?  *Note - 'sugary fizzy drinks' does not include fruit juice.* | 1 – Strongly in favour  2 – In favour  3 – Neutral  4 – Against  5 – Strongly against |
| 40.5 | Reducing the standard size of unhealthy snacks or drinks, like chocolate bars or cans of sugary fizzy drinks? | 1 – Strongly in favour  2 – In favour  3 – Neutral  4 – Against  5 – Strongly against |
| 40.6 | Providing many more free weight management courses for people who want to lose weight? | 1 – Strongly in favour  2 – In favour  3 – Neutral  4 – Against  5 – Strongly against |
| 40.7 | Providing many more free weight management courses for people who want to lose weight? | 1 – Strongly in favour  2 – In favour  3 – Neutral  4 – Against  5 – Strongly against |
| 40.8 | Providing many more operations on the governments’ department of health to help people lose weight - for example, offer minimal invasive weight lose surgery (i.e., fitting gastric band on the stomach to treat obesity and reduce food intake)? | 1 – Strongly in favour  2 – In favour  3 – Neutral  4 – Against  5 – Strongly against |
| 40.8 | Doing more to create or improve cycle paths and pavements to encourage people to be more active? | 1 – Strongly in favour  2 – In favour  3 – Neutral  4 – Against  5 – Strongly against |
| 40.9 | Raising taxes on fuel and parking to encourage people to walk and cycle more? | 1 – Strongly in favour  2 – In favour  3 – Neutral  4 – Against  5 – Strongly against |

| **Section 7: Physical activity** | | |
| --- | --- | --- |
| We are interested in finding out about the kinds of physical activities that people do as part of their everyday lives. The questions will ask you about the time you spent being physically active in the last 7 days. Please answer each question even if you do not consider yourself to be an active person. Please think about the activities you do at work, as part of your house and yard work, to get from place to place, and in your spare time for recreation, exercise, or sport. | | |
| Think about all the **vigorous activities** that you did in the last 7 days. Vigorous physical activities refer to activities that take hard physical effort and make you breathe much harder than normal. Think only about those physical activities that you did for at least 10 minutes at a time. | | |
| 41 | During the last 7 days, on how many days did you do vigorous physical activities like heavy lifting, digging, aerobics, or fast bicycling?  **No vigorous physical activities ***Skip to question 43*** | 42.1____ days per week. |
| 42 | How much time did you usually spend doing vigorous physical activities on one of those days? | 43.1_____ hours per day  43.2_____ minutes per day  43.3 _____ Don’t know/Not sure |
| Think about all the **moderate activities** that you did in the last 7 days. Moderate activities refer to activities that take moderate physical effort and make you breathe somewhat harder than normal. Think only about those physical activities that you did for at least 10 minutes at a time. | | |
| 43 | During the last 7 days, on how many days did you do moderate physical activities like carrying light loads, bicycling at a regular pace, or doubles tennis? Do not include walking.  **No moderate physical activities ***Skip to question 45*** | 43.1_____ days per week. |
| 44 | How much time did you usually spend doing moderate physical activities on one of those days? | 44.1_____ hours per day  44.2_____ minutes per day  44.3 _____ Don’t know/Not sure |
| 45 | During the last 7 days, on how many days did you walk for at least 10 minutes at a time?  **No walking ***Skip to question 47*** | 45.1_____ days per week. |
| 46 | How much time did you usually spend walking on one of those days? | 46.1_____ hours per day  46.2_____ minutes per day  46.3 _____ Don’t know/Not sure |
| The last question is about the time you spent sitting on weekdays during the last 7 days. Include time spent at work, at home, while doing course work and during leisure time. This may include time spent sitting at a desk, visiting friends, reading, or sitting or lying down to watch television. | | |
| 47 | During the last 7 days, how much time did you spend sitting on a weekday? | 47.1_____ hours per day  47.2_____ minutes per day  47.3 _____ Don’t know/Not sure |
